# Supplementary material for: The microbiome biomarkers of pregnant women’s vaginal area predict preterm prelabor rupture in Western China
Source: Front Cell Infect Microbiol. 2024 Oct 31;14:1471027. doi: 10.3389/fcimb.2024.1471027 (PMC11560878; doi:10.3389/fcimb.2024.1471027)
Supplement: Supplementary file 1 [file DataSheet1.zip › compare_1/Community/Structure_Barplot/genus/genus.taxon.Barplot.Legend.With.superkingdom.pdf]

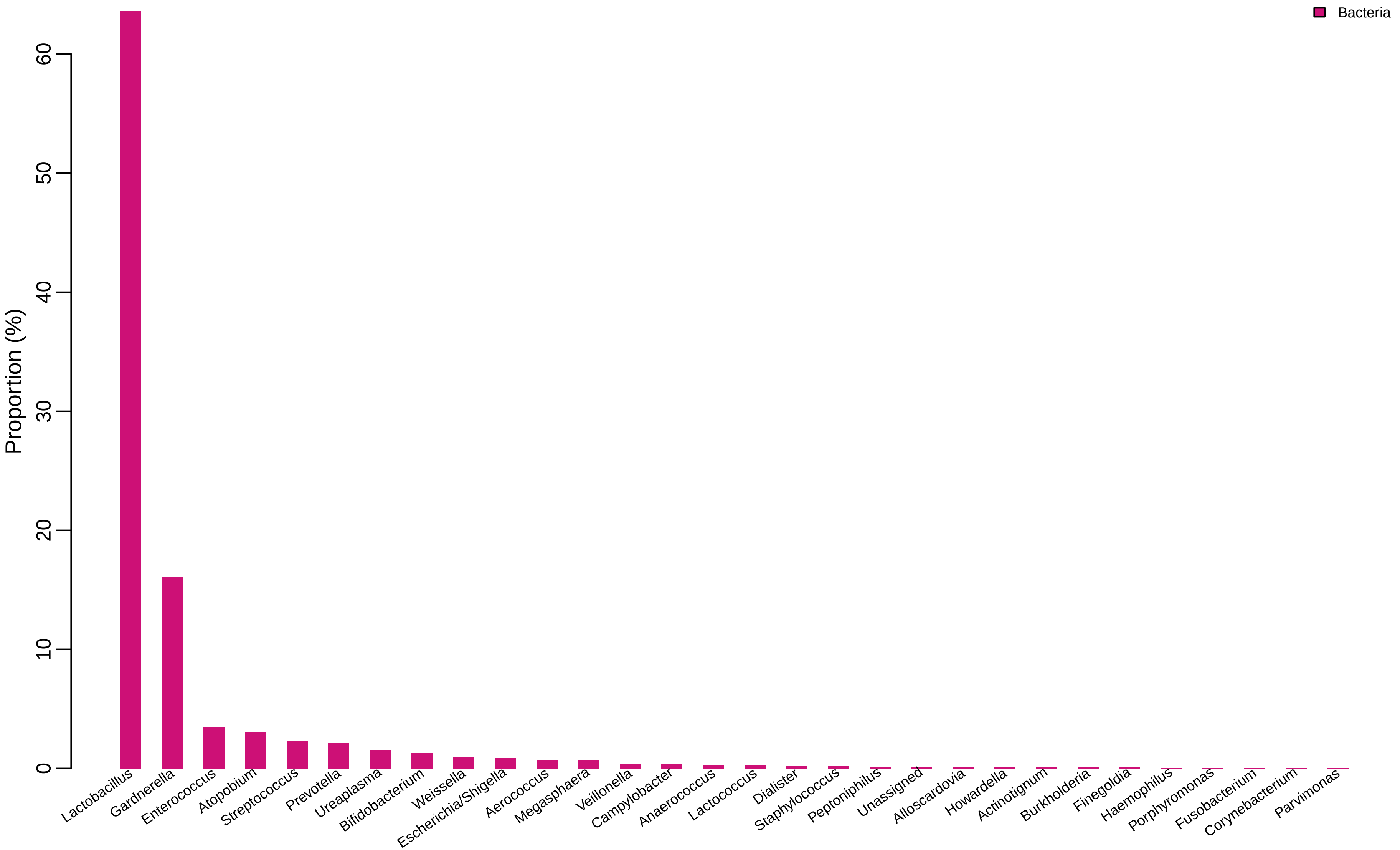

Bacteria
Proportion (%)
0
10
20
30
40
50
60
Lactobacillus
Gardnerella
Enterococcus
Atopobium
Streptococcus
Prevotella
Ureaplasma
Bifidobacterium
Weissella
Escherichia/Shigella
Aerococcus
Megasphaera
Veillonella
Campylobacter
Anaerococcus
Lactococcus
Dialister
Staphylococcus
Peptoniphilus
Unassigned
Alloscardovia
Howardella
Actinotignum
Burkholderia
Finegoldia
Haemophilus
Porphyromonas
Fusobacterium
Corynebacterium
Parvimonas
